# Targeting DEC-205^−^DCIR2^+^ dendritic cells promotes immunological tolerance in proteolipid protein-induced experimental autoimmune encephalomyelitis

**DOI:** 10.1186/s10020-018-0017-6

**Published:** 2018-05-03

**Authors:** Inna Tabansky, Derin B. Keskin, Deepika Watts, Cathleen Petzold, Michael Funaro, Warren Sands, Paul Wright, Edmond J. Yunis, Souhel Najjar, Betty Diamond, Yonghao Cao, David Mooney, Karsten Kretschmer, Joel N. H. Stern

**Affiliations:** 1 Department of Neurobiology and Behavior, The Rockefeller University, New York, NY USA; 2 Department of Medical Oncology, Dana Farber-Harvard Cancer Institute, Boston, MA USA; 3 Departments of Neurology, Surgery, Molecular Medicine, and Science Education, Zucker School of Medicine at Hofstra/Northwell, Hempstead, NY USA; 4 Molecular and Cellular Immunology/Immune Regulation, CRTD/DFG-Center for Regenerative Therapies Dresden, Technische Universität Dresden, Dresden, Germany; 5 Paul Langerhans Institute Dresden, German Center for Diabetes Research (DZD), Dresden, Germany; 6 Department of Autoimmunity, The Feinstein Institute for Medical Research, Northwell Health, Manhasset, NY USA; 7 Department of Engineering, School of Engineering and Applied Sciences, Harvard University, Cambridge, MA USA; 8 Department of Neurology, Lenox Hill Hospital, Northwell Health, New York, NY USA

**Keywords:** Multiple sclerosis, DCIR2, Regulatory T cells, PLP139–151, T cells, Dendritic cells

## Abstract

**Background:**

Dendritic cells (DC) induce adaptive responses against foreign antigens, and play an essential role in maintaining peripheral tolerance to self-antigens. Therefore they are involved in preventing fatal autoimmunity. Selective delivery of antigens to immature DC via the endocytic DEC-205 receptor on their surface promotes antigen-specific T cell tolerance, both by recessive and dominant mechanisms. We provide evidence that the induction of antigen-specific T cell tolerance is not a unique property of CD11c^+^CD8^+^DEC-205^+^ DCs.

**Methods:**

We employed a fusion between αDCIR2 antibodies and the highly encephalitogenic peptide 139–151 of myelin-derived proteolipid protein (PLP_139–151_), to target CD11c^ +^CD8^-^ DCs with a DEC-205−DCIR2^+^ phenotype in vivo, and to substantially improve clinical symptoms in the PLP_139–151_-induced model of experimental autoimmune encephalomyelitis (EAE).

**Results:**

Consistent with previous studies targeting other cell surface receptors, EAE protection mediated by αDCIR2-PLP_139–151_ fusion antibody (Ab) depended on an immature state of targeted DCIR2^+^ DCs. The mechanism of αDCIR2-PLP_139–151_ mAb function included the deletion of IL-17- and IFN-γ-producing pathogenic T cells, as well as the enhancement of regulatory T (Treg) cell activity. In contrast to the effect of αDEC-205^+^ fusion antibodies, which involves extrathymic induction of a Foxp3^+^ Treg cell phenotype in naïve CD4^+^Foxp3^-^ T cells, treatment of animals with DCIR2^+^ fusion antibodies resulted in antigen-specific activation and proliferative expansion of natural Foxp3^+^ Treg cells.

**Conclusions:**

These results suggest that multiple mechanisms can lead to the expansion of the Treg population, depending on the DC subset and receptor targeted.

**Electronic supplementary material:**

The online version of this article (10.1186/s10020-018-0017-6) contains supplementary material, which is available to authorized users.

## Background

Experimental autoimmune encephalomyelitis (EAE) is a disease that can be induced by several methods, and it is a widely accepted mouse model for the human disease multiple sclerosis (MS). MS is characterized demyelination of axons in the central nervous system (CNS) due to autoimmune attack (Tabansky et al., 2015). Mice with EAE display acute and chronic inflammation and demyelination similar to MS patients (Raine et al., 1980). In mice, EAE results in progressive paralysis that begins at the tip of the tail and moves forward along the body. The most common method of EAE induction involves injection of antigenic protein fragments of myelin, along with an adjuvant. Recognition and uptake of these protein fragments by dendritic cells (DC) are necessary for the subsequent inflammatory response to myelin (Izikson et al., 2000). While DC comprise the smallest population of leukocytes, they appear to be the principle antigen presenting cells (APCs) of the immune system (Jung et al., 2002; Steinman & Witmer, 1978). DC play a key role in activating naïve T cells, including autoreactive T cells that escape selection in the thymus (Serafini et al., 2000). They also regulate the inflammatory response in EAE by presenting antigen to T cells (Serafini et al., 2000; Dittel et al., 1999).

While T cells are thought to be the primary effectors of CNS damage in autoimmune disease, they interact and communicate with various cell types, including DC and B cells (Stern et al., 2014). Shortly after injection with myelin protein fragments and adjuvant to induce EAE, an influx of DC into the spinal cord and surrounding regions can be observed (Izikson et al., 2000; Greter et al., 2005; McMenamin, 1999; Bailey et al., 2007; Lande et al., 2008). This increase in cell numbers—which has been proposed to be due to in situ differentiation (Gottfried-Blackmore et al., 2009)—is thought to indicate an ongoing immune response targeting the myelin sheath of neurons.

The target of the fusion antibody in the present study was Dendritic cell inhibitory receptor 2 (DCIR2), which is a transmembrane protein with a single C-type II lectin domain (Nussenzweig et al., 1982; Steinman et al., 1983; Dudziak et al., 2007). DCIR2’s ligands are thought to be biantennary complex-type glycans containing bisecting N-acetylglucosamine (GlcNAc) (Nagae et al., 2013). It is expressed primarily on CD8- DC, which function in Major histocompatibility complex (MHC) class II antigen presentation to induce maturation of helper T cells (Th cells) (Dudziak et al., 2007; Kasahara & Clark, 2012). In the absence of maturation stimuli, these DC are capable of stimulating natural Foxp3+ regulatory T cells (Treg) and mediating tolerance to self-antigens (Yamazaki & Morita, 2013; Yamazaki et al., 2008). However, antigen delivered with an adjuvant or another maturation stimulus leads to T cell expansion and production of pro-inflammatory cytokines, such as IFN-γ (Kool et al., 2008; Sharp et al., 2009). Inflammatory autoimmune demyelinating diseases in mammals are thought to involve the activation of Th1 and Th17 autoreactive T cells that secrete the pathogenic interleukins IFN-γ and IL-17 (Bettelli et al., 2004; Korn et al., 2009; Ghoreschi et al., 2010). In the case of EAE, autoreactive T cells recognize the myelin protein fragment presented on the class II MHC molecules of DC, along with costimulatory molecules such as CD80 and CD86. This activates the T cells instead of promoting tolerance, resulting in an autoimmune attack on the myelin surrounding the nerves in the CNS (Guidetti et al., 2001).

Several previous studies have indicated that targeting DCs with αDEC-205 fused to MOG_35–55_ (myelin oligodendrocyte glycoprotein, amino acids 35–55), results in the amelioration of EAE in the MOG_35–55_ mouse model (Idoyaga et al., 2013). αDEC-205 is a monoclonal antibody specific to CD8+ DC, and MOG_35–55_ is a minor component of myelin employed in the induction of EAE in C57/BL6 mice. DEC-205 is co-expressed with Langerin-1 on conventional CD11c^+^CD8^+^DCIR2^−^ DCs, and some migratory DC subsets such as Langerhans cells and CD103^+^ DCs. Different mechanisms have been proposed for how DC targeting causes amelioration of EAE. Initially, deletion or anergization of autoreactive T cells was emphasized (Stern et al., 2010), but subsequent experiments have revealed a prominent role for generation of Tregs, resulting in antigen-specific tolerance. Thus, multiple mechanisms of tolerance induction can account for the effectiveness of the αDEC-205 fusion mAb (Petzold et al., 2012).

Several EAE models exist, including the PLP_139–151_ model in SJL/J mice and the MOG_35–55_ in Bl6/DBA mice. In each of the models, EAE can be induced via the delivery of the appropriate peptide in synchrony with Complete Freud’s Adjuvant (CFA). Previous studies have indicated that in the MOG_35–55_ model, αDEC-205-MOG_35–55_ fusion antibody treatment worked by two distinct mechanisms: T cell anergy and CD5-mediated conversion of T regs (Hawiger et al., 2004). In addition to examining whether the targeting of a different DC subtype would also ameliorate the symptoms of EAE, we were curious to investigate whether the effects of DC targeting on the immune systems are conserved between mouse models of EAE. We therefore opted to use the SJL/J PLP_139–151_ model because PLP is highly abundant in the CNS and the model has clinical features that have been reported to be similar to MS (Miller & Karpus, 2007). We have previously used the SJL/J PLP_139–151_ model to characterize the αDEC-205-PLP_139–151_ mAb mediated protection against EAE (Stern et al., 2010), and the more detailed mechanistic analysis presented here will enable us to compare the effect in the SJL/J model against existing C57/Bl6 literature (Miller & Karpus, 2007).

In the SJL/J model, we addressed three separate functional and mechanistic issues. First, we investigated whether using the αDCIR2 fusion antibody (Nussenzweig et al., 1982; Dudziak et al., 2007) to target a different subset of DC (separate from subset targeted by αDEC-205) will also result in EAE amelioration. Thus, we targeted CD11b^+^ DCs, which in their mature state have recently been proposed to be involved in the generation of memory T cells, as opposed to effector cells (Kim et al., 2014). We further sought to compare the effect of pretreatment with each of the two different fusion antibodies (αDCIR2 and αDEC-205) on the immune system during active disease. Finally, we sought to investigate whether the T regs induced by antibody treatment would confer a protective effect in EAE.

## Methods

### Mice

SJL/J mice (H-2^s^) were purchased from The Jackson Laboratory (Bar Harbor, Maine, USA) and maintained at the animal facilities of Harvard University according to the animal protocol guidelines of Harvard University and Harvard Medical School. Thy-1.2 BALB/c mice, congenic Thy1.1 BALB/c.Foxp3^IRES-GFP^ mice (Haribhai et al., 2007) with expression of a transgenic TCR reactive to the influenza haemagglutinin peptide 109–117 (TCR-HA_109–117_), and Thy1.1 BALB/c.Foxp3^IRES-GFP^ x TCR-HA_109–117_ mice that additionally express the HA protein under control of a ubiquitous phosphoglycerate kinase promoter (Pgk-HA) (Klein et al., 2003) were bred and maintained under specific pathogen-free conditions at the Experimental Center of the Medical Theoretical Center (Dresden University of Technology, Dresden, Germany). All animal experiments that involved Thy-1.2 BALB/c and Thy1.1 BALB/c.Foxp3^IRES-GFP^ x TCR-HA_109–117_ mice with or without additional Pgk-HA expression were performed as approved by the Regieriungspräsidium Dresden (Dresden Germany).

### Recombinant fusion antibody production

Eukaryotic expression vectors encoding the IgH and IgL chain cDNA of cloned αDEC-205 (NLDC-145), αDCIR2 and the respective isotype control mAbs were produced in the Nussenzweig laboratory (Rockefeller University, New York, USA). For the generation of recombinant mAbs fused to peptide antigens of interest, we first constructed double-stranded DNA fragments coding for either PLP_139–151_ or HA_109–117_ with spacer residues on both sides using synthetic oligonucleotides, which were then added in frame to the C terminus of the cloned IgH chain encoding cDNA, as described before (Kretschmer et al., 2006). Recombinant mAbs were produced, using the FreeStyle™ 293 Expression System (Invitrogen), as described previously (Kretschmer et al., 2006; Petzold et al., 2010). In brief, HEK-293 cells were grown as suspension cultures in serum-free FreeStyle™ 293 medium and transiently co-transfected with the respective IgH and IgL chain plasmids using FreeStyle™ MAX reagent. The produced mAbs were purified on HiTrapTM Protein G HP columns (GE Healthcare).

### Effect of αDCIR2-PLP_139–151_ fusion mAbs on the induction of EAE

To determine the therapeutic effect of DCIR2^+^ DC targeting on the clinical outcome of EAE, cohorts of 6 to 10-week-old female SJL/J mice were either left untreated or injected i.p. with 1μg of fusion mAbs (αDCIR2-PLP_139–151_ mAb, isotype control/ PLP_139–151_ mAb) 10 days before inducing EAE. Where indicated, cohorts of control mice were injected with 1 μg of conventional αDCIR2 mAb. For EAE induction, SJL/J females were immunized s.c. with 75 μg of synthetic PLP_139–151_ peptide emulsified in CFA. Pertussis toxin (200 ng, List Biological Laboratories, Campbell, CA) was administered i.v. on the next day. The mice were regularly monitored for appearance of clinical signs of EAE (clinical score: 0–5: 1, limp tail; 2, hind limb paralysis; 3, complete hind limb paralysis; 4, four limbs paralyzed; 5, moribund).

### Cell sorting and flow cytometry

Single cell suspensions of spleen, mesenteric lymph nodes (mLNs), or various subcutaneous lymph nodes (scLN) (Schallenberg et al., 2010) were prepared using 70 μm cell strainers (BD Biosciences). mAbs to CD4 (RM4–5, GK1.5), CD25 (PC61, 7D4), CD62L (MEL-14) and Thy1.1 (OX-7), as well as Fc receptor–blocking mAbs to CD16/32 (93) and Pacific Blue- and APC-conjugated streptavidin were obtained from eBioscience or BD Biosciences. The mAb to TCR-HA_107–119_ (6.5) was purified and conjugated to Alexa Fluor 647 (Invitrogen) in our laboratory according to standard protocols. Before FACS, for some experiments, CD4^+^ CD25^+^ cells were enriched using biotinylated mAbs against CD4 or CD25, respectively, streptavidin-conjugated microbeads and the AutoMACS (Miltenyi Biotec). Samples were analyzed on a LSR II or sorted using a FACSAria II or III (BD). Data were analyzed using FlowJo (Tree Star, Inc.).

### Adoptive cell transfers and mechanistic studies on DC targeting

To assess the relative contribution of dominant tolerance to DCIR2^+^ DC-mediated protection from EAE, cohorts of SJL/J mice were pre-treated with either recombinant αDCIR2-PLP_139–151_ mAb or conventional αDCIR2 mAb (1 μg per mouse). EAE was induced as described above. At day 10 after EAE induction, the spleens were harvested and 5 × 10^6^ total splenocytes were i.v. injected into naïve 6–8-week-old SJL/J females. After EAE induction, the recipient mice were scored daily for clinical signs of EAE (see above for details). For mechanistic studies, CD4^+^TCR-HA^+^ T cells with a naïve (CD4^+^TCR-HA^+^ CD62L^high^CD25^−^Foxp3^IRES-GFP−^) and a Foxp3^+^ Treg cell (CD4^+^TCR-HA^+^CD25^+^Foxp3^IRES-GFP+^) phenotype were isolated by FACS from peripheral lymphoid tissues (pooled spleen and LNs) of Thy1.1 BALB/c.Foxp3^IRES-GFP^ x TCR-HA_109–117_ mice and Thy1.1 BALB/c.Foxp3^IRES-GFP^ x TCR-HA_109–117_ x Pgk-HA mice, respectively. Prior to i.v. injection into Thy1.2 BALB/c recipient mice, highly pure populations of sorted CD4^+^TCR-HA^+^ T cells were labeled with 20 μM eFluor670. The next day, recipient mice were either left untreated, or injected with indicated amounts (100 ng, 1 μg) of recombinant fusion mAbs (αDEC-205-HA_109–117_, αDCIR2-HA_109–117_). On days 7 and 14 after adoptive T cell transfer and recombinant fusion mAb administration, congenic Thy1.1^+^CD4^+^TCR-HA^+^ T cells in peripheral lymphoid tissues (spleen, mLNs, scLNs) of Thy1.2 recipient mice were analyzed by flow cytometry for Foxp3 expression and cell division, as judged by eFluor670 dilution.

### IL-17 and IFN-γ ELISpot assay

10 days after EAE induction, the fraction of cytokine-producing cells among total splenocytes was determined, either by using a mouse IL-17 ELISpot kit (R&D Systems) according to the manufacturer’s protocol, or by performing IFN-γ ELISpot assays as described previously (Stern et al., 2010). All ELISpots assay plates were counted using an Immunospot™ counter.

### Statistical analysis

Differences between mean EAE scores were analyzed by Student’s t-test. Elispot results were analyzed by the Dunnett’s comparisons test.

## Results

### Preimmunization with αDCIR2-PLP_139–151_ mAb reduces disease severity of EAE induced with PLP_139–151_

**Fig. 1:**
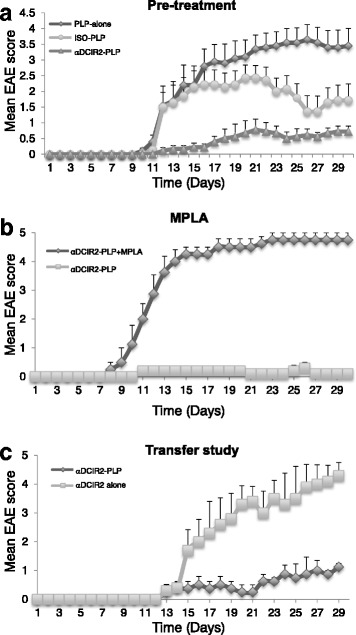
Preimmunization with αDCIR-PLP_139–151_ fusion antibodies (Abs) ameliorates EAE (PLP_139–151_ abbreviated in the figure as PLP due to space concerns). **a** Preimmunization with αDCIR2-PLP_139–151_ mAb (αDCIR2-PLP) ameliorates EAE in SJL/J mice. Pretreatment with the αDCIR2-PLP_139–151_ mAb prior to disease induction decreased severity of EAE in mice. Mice were injected i.p. with 1 μg of fusion antibodies (either αDCIR2-PLP_139–151_ mAb or ISO-PLP_139–151_ mAb (ISO-PLP) 10 days prior to disease induction (labeled as − 10), and EAE was induced starting on day 1. Mice were monitored daily for clinical signs of EAE, and disease severity was scored. Mean EAE scores for mice in each group (*n* = 5) are shown. Disease severity was decreased, and disease onset was delayed in mice that had been preimmunized with αDCIR2-PLP_139–151_ mAb. Mice that received αDCIR2-PLP_139–151_ mAb had significantly lower disease scores compared to controls that had not been pre-treated with any mAbs. Significant reduction of disease was observed on days 12 to 19 (*p* < 0.003) and days 22 to 30 (*p* < 0.009). Mice treated with DCIR2-PLP_139–151_ were also significantly different from control mice treated with ISO-PLP_139–151_ (*p* < 0.02 from days 12 to 17 and 24 to 28). The data presented represent three pooled independent experiments. **b** Using MPLA to induce maturation of DC concurrent with mAb administration abrogated the protective effect of preimmunization with DCIR2-PLP_139–151_. MPLA (10 μg) was co-administered with either αDCIR2-PLP_139–151_ mAb (*n* = 5) or ISO-PLP_139–151_ 10 days before induction of EAE. To induce EAE, mice were injected with PLP_139–151_ in CFA. Pertussis toxin (PT) (200 ng) was administered into the tail vein the following day. Disease progression in mice that received MPLA with αDCIR2-PLP_139–151_ mAb was not significantly different from control mice treated with ISO/PLP_139–151_ mAb and MPLA (*p* > 0.05). **c** Adoptive transfer of splenocytes from mice treated with αDCIR2-PLP_139–151_. SJL/J mice were preimmunized i.p. with 1 μg of either αDCIR2-PLP_139–151_ mAb or αDCIR2 mAb alone ten days prior to induction of EAE. To induce disease, mice were immunized with 75 μg s.c. of PLP_139–151_ peptide, and 200 ng of pertussis toxin (pt) i.v. the next day. Splenocytes (5 × 10^6^) were isolated from these animals 10 days after disease induction and injected intravenously into naïve SJL/J mice along with 75 μg s.c. of PLP_139–151_ in CFA, followed by PT (200 ng i.v.) the next day. Mice were monitored daily for clinical signs of EAE and disease severity was scored. Mean EAE scores mice in each group (*n* = 5) are shown. Mice that received splenocytes from animals that had been treated with αDCIR2-PLP_139–151_ mAb had significantly lower disease scores compared to recipients of spenocytes from control animals treated with αDCIR2 mAb alone. The difference in disease scores was observed days 15, 20–24 and 28 to 29 (*p* < 0.02, Student’s t-test). The experiment was repeated several times with similar results; a representative experiment is shown

To determine whether targeting DCIR2 could induce immune tolerance, we tested whether preimmunization with the αDCIR2-PLP_139–151_ fusion mAb improves clinical symptoms in the SJL/J mouse model of PLP_139–151_-induced EAE. Prior to EAE induction, cohorts of female SJL/J mice were either left untreated (no preimmunization), received a single dose injection of 1 μg αDCIR2-PLP_139–151_ mAb, or equivalent amounts of a recombinant isotype control mAb fused to thePLP_139–151_ peptide (ISO-PLP_139–151_). After 10 days, EAE was induced with 75 μg of free synthetic PLP_139–151_ peptide in Complete Freund’s Adjuvant (CFA), along with 200 ng of pertussis toxin (PT) administered the following day. Mice were scored for clinical symptoms every day after disease induction. Overall, and as compared to control groups (no preimmunization, ISO-PLP_139–151_), αDCIR2-PLP_139–151_ pre-administration resulted in a substantial delay in disease onset and a marked reduction in disease severity, as revealed by mean maximum EAE scores (Fig. 1a). All mice in the control cohorts developed clinical signs of EAE by day 10 after disease induction, whereas EAE symptoms were not observed in mice that had been preimmunized with αDCIR2-PLP_139–151_ mAb until day 18. In addition, disease severity was significantly reduced (*p* < 0.003, Student’s t-test on multiple days, Fig. 1a) in mice that had been treated with αDCIR2-PLP_139–151_ mAb, as compared to the control groups, which had not been preimmunized or which received the isotype control antibody, ISO-PLP_139–151_. Notably, the slight amelioration that was observed in the mice treated with ISO-PLP_139–151_ was consistent with previously published findings using other isotype controls (Stern et al., 2010). As this first experiment indicated that there is a dramatic and statistically significant difference between mice treated with ISO-PLP_139–151_ and αDCIR2-PLP_139–151_ mAbs, we opted not to use the isotype control in subsequent experiment, partly due to animal welfare concerns. In the control groups, one mouse died shortly after disease induction, and the remaining mice entered a chronic phase of the disease, with a mean maximum score of 3 on day 14. In contrast, the maximum mean score of αDCIR2-PLP_139–151_ mAb treated mice was 1.2 (tail paralysis) on day 20 (Fig. 1a). No mortalities occurred in any of the mice preimmunized with the αDCIR2-PLP_139–151_ mAb.

### Steady-state DC are required for EAE protection conferred by αDCIR2-PLP_139–151_ mAb treatment

Current thinking suggests that immature DC are required for immunization with fusion mAbs to be able to protect against disease. To determine immature DC also mediate the function of αDCIR2-PLP_139–151_, co-administered the antibody with the toll-like receptor 4 (TLR) ligand monophospholipid A (MPLA): a low-toxicity derivative of LPS with potent proinflammatory activity that induces maturation and activation of immature DCs. Ten days prior to disease induction, we preimmunized a cohort of initially naïve SJL/J mice with αDCIR2-PLP_139–151_ mAb (1 μg) and MPLA, while SJL/J mice injected with 1 μg αDCIR2-PLP_139–151_ mAb only were used as a control. EAE was induced in both groups of mice by administration of PLP_139–151_/CFA and PT. Strikingly, 80% of SJL/J mice that had been preimmunized with αDCIR2-PLP_139–151_ mAb/MPLA died by day 15 after EAE induction, resulting in a mean maximum score of 4.7 (Fig. 1b). The rapid progressive paralysis exhibited by these animals was consistent with EAE as the cause of death. In contrast, control mice that received αDCIR2-PLP_139–151_ mAb only exhibited a mean maximal score of only 0.5 (tail paralysis) and no mortality (Fig. 1b). Thus, the maturation and/or activation of DCs by MPLA completely abrogates DCIR2^+^ DC-targeted protection from EAE.

### Adoptive transfer of splenocytes mice preimmunized with αDCIR2-PLP_139–151_ mAb reduces disease severity in recipients

DCIR2+ DC have previously been documented to induce expansion of T regs to produce a dominant mechanism of disease suppression (Yamazaki et al., 2008). If this is the case, than transfer of immune cells from mice immunized with αDCIR2-PLP_139–151_ mAb into naïve congenic recipients should confer protection against EAE. To test whether this is the case, cohorts of SJL/J mice were preimmunized with 1 μg of either recombinant αDCIR2-PLP_139–151_ fusion mAb or conventional (unconjugated to peptide) αDCIR2 mAb 10 days before induction of EAE. At day 10 after EAE induction (20 days total after immunization), single cell suspensions were prepared from the spleens of both cohorts, and 0.5–1 × 10^7^ splenocytes were injected i.v. into naïve SJL/J mice. EAE was induced in transfer recipients the next day. As expected, all mice that received splenocytes from unconjugated αDCIR2 mAb-treated donor mice developed severe EAE with high clinical scores (Fig. 1c). In contrast, mice that had received splenocytes from αDCIR2-PLP_139–151_ fusion mAb-pretreated donor mice exhibited substantially lower disease scores at all time points analyzed.

### Pretreatment with αDCIR2-PLP_139–151_ mAb reduces activity of pathogenic T helper cells

**Fig. 2:**
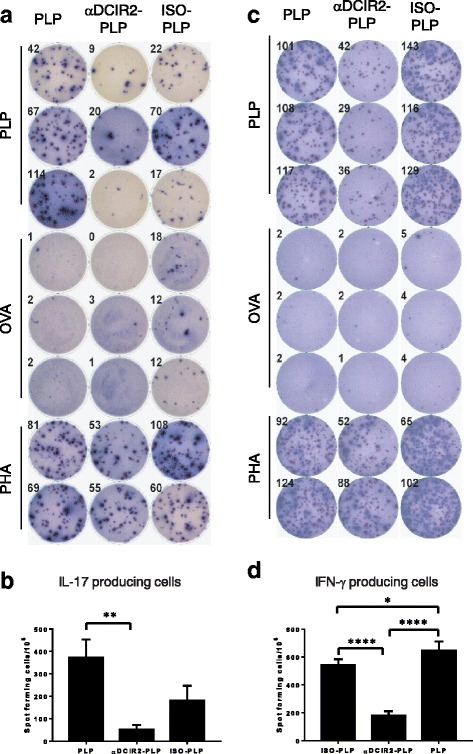
The numbers of pathogenic Th17 (IL-17 producing) and IFN-γ producing cells are significantly reduced in mice treated with αDCIR2-PLP_139–151_ mAb compared to controls (PLP_139–151_ abbreviated in the figure as PLP due to space concerns). **a** Elispot analyses of the impact of preimmunization with αDCIR2-PLP_139–151_ mAb on Th17 cells. SJL/J mice were preimmunized with a low dosage (1 μg) of different fusion antibodies. Ten days later, mice were immunized with PLP_139–151_ and injected i.v. with pertussis toxin (200 ng) the following day to induce EAE. IL-17 ELISpot analyses were conducted on splenocytes isolated from mAb treated and untreated mice 10 days after disease induction. Splenocytes were plated onto IL-17 pre-coated plates and stimulated with 10 μg/ml PLP_139–151_. Wells stimulated with PHA and unstimulated wells were used as controls. Analysis was conducted using an E-biosciences IL-17 ELISpot kit. **b** Quantification of results of the IL-17 ELISpot assay. Pre-immunization with αDCIR2-PLP_139–151_ mAb (*n* = 2, *p* = 0.0059) 10 days before disease induction resulted in a decreased number of cells producing IL-17, as compared to mice that did not receive any mAbs (labeled in the figure as PLP). Number of spots per million cells was calculated by multiplying the average of triplicate wells (2 × 10^5^) by fivefold. **c** IFN-γ ELISPOT analysis on splenocytes isolated from mice treated with mAb treated and untreated controls 10 days after disease induction. Splenocytes were plated onto plates pre-coated with IFN-γ and stimulated with 10 μg/ml PLP_139–151_. PHA and unstimulated wells were used as controls. Analysis was conducted using an IFN-γ ELISpot kit. **d** Quantification of results of the IFN-γ ELISpot assay. Pre-immunization with αDCIR2-PLP_139–151_ mAb (*n* = 2) resulted in a decreased number of IFN-γ producing cells, as compared to mice that had been preimmunized with ISO-PLP_139–151_ mAb (*n* = 2, *p* = 0.0001) or not preimmunized with either mAb (*n* = 2, p = 0.0001). Notably, consistent with the slight disease amelioration seen in Fig. 1, pre-treatment with ISO-PLP_139–151_ mAb also resulted in a reduction of IFN-γ producing cells as compared to PLP-treated mice (*n* = 2, *p* = 0.0124). Number of spots per million cells was calculated by multiplying the average of triplicate wells (2 × 10^5^) splenocyes per well) by fivefold

Another potential mechanism for induction of immunological tolerance is the deletion or anergy of pathogenic T cells. To determine whether this mechanism contributes to EAE protection induced by preimmunization with antibodies, assessed the impact of tolerogenic DCIR2^+^ DC vaccination on the activity of encephalitogenic T helper (Th) cells. For this experiment, cohorts of SJL/J mice were either preimmunized with recombinant αDCIR2-PLP_139–151_ or ISO-PLP_139–151_ mAbs (1 μg/mouse), or were left untreated. Ten days post preimmunization, EAE was induced in all groups of mice by administration of PLP_139–151_/CFA and PT. Total splenocytes were isolated 10 days after EAE induction and the number of PLP_139–151_-reactive Th cells secreting IL-17 and IFN-γ was quantified by the ELISPOT assay (Fig. 2). Consistent with the slight EAE amelioration observed in this treatment group, pre-administration of the ISO-PLP_139–151_ mAb resulted in somewhat decreased numbers of Th cells secreting cytokines, as compared to control mice that were not preimmunized (Fig. 2a-d). While the decrease in IL-17^+^ Th cells in ISO-PLP_139–151_ treated mice was not significant compared to controls, the decrease in IFN-γ^+^ secreting Th cells was (*p* < 0.02). In contrast, preimmunization with αDCIR2-PLP_139–151_ caused significant decreases the numbers of IL-17^+^ Th cells as compared to un-preimmunized controls (*p* = 0.0059, Fig. 2a, b) and a highly significant decrease in IFN-γ^+^ secreting Th cells (*p* = 0.0001, Fig. 2c, d), as compared to both control groups. Thus, αDCIR2-PLP_139–151_ mAb administration prior to EAE induction results in a substantial reduction in encephalitogenic Th cell activity, possibly due to deletion and/or induction of an anergic state in PLP_139–151_-reactive Th cells. Alternatively, DCIR2^+^ DC targeting may promote the antigen-specific suppressor activity of Foxp3^+^ Treg cells.

### Tracking antigen-specific CD4^+^ T cells upon antigen targeting of steady-state DEC-205^+^ and DCIR2^+^ DC illustrates mechanistic differences between the function of the two antibodies

The results of the experiments described above suggested that tolerogenic αDCIR2^+^-PLP_139–151_ fusion mAb vaccination improves the outcome of PLP_139–151-_induced EAE both by recessive (depletion/induction of anergy in pathogenic T cells, Fig. 2) and dominant (enhancement of Treg cell activity, Fig. 1c) mechanisms of immunological tolerance. To obtain further mechanistic insight, we tracked changes in numbers of antigen-specific CD4^+^ T cells after selective delivery of antigen either to DEC-205^+^ or to DCIR2^+^ DCs in the steady state. For this experiment, we took advantage of a well-established TCR/agonist ligand transgenic model system that can be used as a source of truly naïve CD4^+^Foxp3^−^ T cells and CD4^+^Foxp3^+^ Treg cells with the same antigen specificity (Klein et al., 2003; Hinterberger et al., 2010; D'Cruz & Klein, 2005). In peripheral lymphoid tissues of these mice, which have transgenic expression of TCR-HA_109–117_, CD4^+^TCR-HA^high^ T cells exhibit a naïve CD62L^+^CD25^−^Foxp3^−^ phenotype, due to the absence of the TCR agonist ligand (Additional file 1: Figure S1A). In contrast, in double-transgenic mice that co-express the TCR-HA_109–117_ on CD4^+^ T cells and the HA protein under control of the ubiquitous phosphoglycerate kinase promoter (Pgk-HA), the neo-self-antigen HA promotes intrathymic induction and peripheral accumulation of TCR-HA^+^Foxp3^+^ Treg cells (Additional file 1: Figure S1B) (Klein et al., 2003; Hinterberger et al., 2010; D'Cruz & Klein, 2005).

**Fig. 3:**
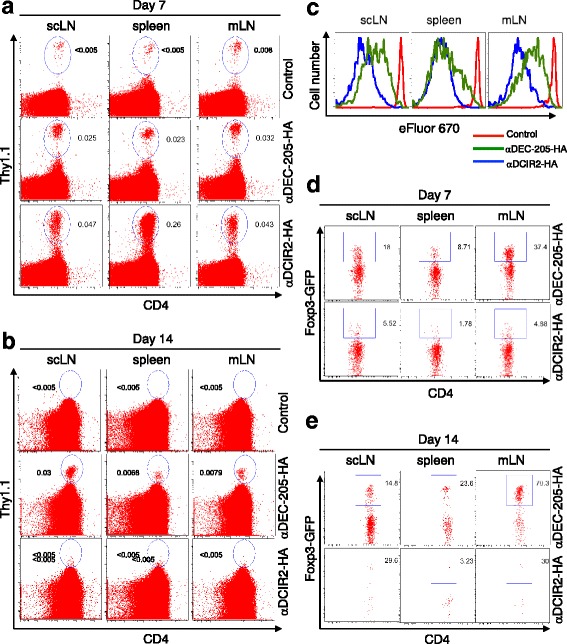
Effect of preimmunization with αDCIR2-HA_109–117_ on Thy1.1^+^ and Foxp3^+^ cells (HA_109–117_ abbreviated in the figure as HA due to space concerns). Naïve TCR transgenic CD4 T cells recognizing HA_109–117_ peptide were injected into immunocompetent congenic recipients, which were subsequently injected with either αDEC-205-HA_109–117_ or αDCIR2-HA_109–117_ fusion mAb. In both, **a** cases antigen-specific Thy1.1+ T cells can be tracked in various peripheral lymphoid organs, and **c** αDCIR2 immunization results in somewhat increased proliferation. **b** However, on day 14, essentially all Thy1.1^+^ T cells appeared to be deleted in mice immunized with αDCIR2-HA, whereas significant populations of Thy1.1^+^ T cells could still be detected in mice that had received the same amount of αDEC-205-HA_109–117_ fusion mAb. Additionally, immunization with αDEC-205-HA_109–117_ mAb results in increased Foxp3 expression, as detected on **d** day 7 and **e** day 14. In contrast, immunization with αDCIR2-HA_109–117_ mAb leads to a marginal increase in Foxp3 expression on **d** day 7, and **e** efficient deletion of all cells on day 14

To test how CD4+ T cells respond to treatment with different fusion mAbs, we adoptively transferred small numbers of FACS-purified populations of naïve CD4^+^Foxp3^−^TCR-HA^+^ T cells into fully immunocompetent recipient mice. These mice were either left untreated or injected with recombinant αDEC-205-HA_109–117_ or αDCIR2-HA_109–117_ fusion mAbs post-transfer (Fig. 3). In untreated CD4^+^Foxp3^−^TCR-HA^+^ T recipient mice, congenic marker^+^ CD4^+^TCR-HA^+^ T cells were still detectable in the peripheral lymphoid tissues on day 7 after adoptive transfer (Fig. 3a), but were below the level of detection on day 14 (Fig. 3b). As expected (Kretschmer et al., 2006; Schallenberg et al., 2010; Kretschmer et al., 2005), a single dose injection of 100 ng αDEC-205-HA_109–117_ mAb resulted in moderate proliferation (Fig. 3a-c) and concomitant induction of Foxp3 expression in a significant proportion of initially naïve CD4^+^Foxp3^−^TCR-HA^+^ T cells (Fig. 3d, e). In contrast, upon administration of equivalent amounts of αDCIR2-HA_109–117_ fusion mAb, CD4^+^TCR-HA^+^Thy1.1^+^ T cells vigorously proliferated and exhibited only marginal Foxp3 expression at day 7 after transfer (Fig. 3a, c). This proliferation was followed by efficient deletion of essentially all cells by day 14 after adoptive transfer (Fig. 3b, e).

**Fig. 4:**
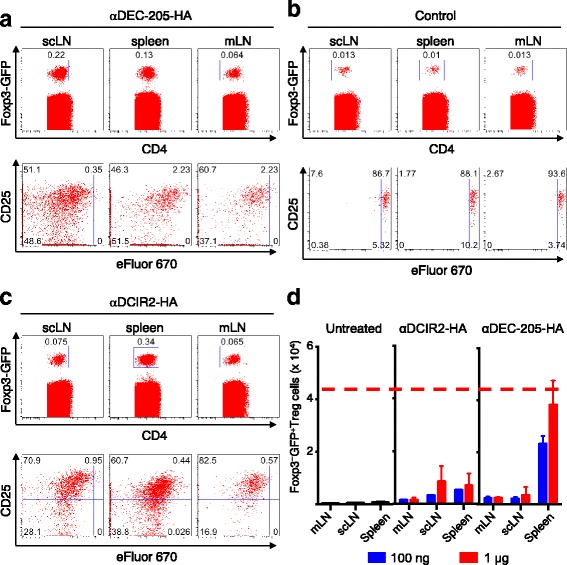
Effect of immunization with αDEC-205-HA_109–117_ on populations of CD4^+^CD25^+^Foxp3^+^ Treg cells (HA_109–117_ abbreviated in the figure as HA due to space and readability concerns). Small numbers of TCR transgenic CD4 + CD25 + Foxp3+ Treg cells that recognize an HA peptide were injected into immunocompetent congenic recipients, which were subsequently injected with either αDEC-205-HA_109–117_ or αDCIR2-HA_109–117_ fusion mAbs. **a** FACS plot of Foxp3 (GFP), CD25 and CD4 expression in cells isolated from spleens, mesenchymal (mLN), and supraclavicular (scLN) lymph nodes of mice treated with αDEC-205-HA_109–117_ mAb. **b** FACS plot of Foxp3 (GFP), CD25 and CD4 expression in cells isolated from spleens, and mesenchymal and supraclavicular lymph nodes of control mice. In untreated mice, congenic marker positive Treg cells that can be tracked in peripheral lymphoid tissues maintain Foxp3 and CD25 expression but do not undergo proliferation (day 6 after injection). **c** FACS plot of Foxp3 (GFP), CD25 and CD4 expression in cells isolated from spleens and mesenchymal and supraclavicular lymph nodes of mice treated with αDCIR2-HA_109–117_ mAb. **d** Histogram of results from analyses shown in (**a**-**c**). Both αDEC-205-HA_109–117_ and αDCIR2-HA_109–117_ fusion mAbs promote proliferative expansion of antigen-specific Treg cells. Increases in the number of Foxp3-GFP^+^ Treg cells is observed after treatment with αDCIR2-HA_109–117_ fusion antibodies

Next, we assessed the impact of the two antibodies on the maintenance, activation and proliferative expansion of pre-formed CD4^+^Foxp3^+^ Treg cells. To this end, we injected FACS-purified Foxp3^+^TCR-HA^+^ Treg cells (CD4^+^CD25^+^TCR-HA^+^Foxp3^IRES-GFP+^) from the peripheral lymphoid tissues of TCR-HA_109–117_ x Pgk-HA mice (Additional file 1: Figure S1B) into fully immunocompetent, congenic recipient mice. Subsequently, the recipients were either left untreated or injected with recombinant αDEC-205-HA_109–117_ or αDCIR2-HA_109–117_ fusion mAbs (Fig. 4). In peripheral lymphoid tissues of untreated recipients, small populations of congenic marker^+^ TCR-HA^+^ Treg cells could be tracked 6 days after adoptive transfer. These cells did not undergo proliferation in the absence of cognate antigen, but maintained Foxp3 and CD25 expression (Fig. 4). In contrast, treatment with both αDEC-205-HA_109–117_ mAb (Fig. 4a) and αDEC-205-HA_109–117_ mAb (Fig. 4c) targeting of HA_109–117_ induced substantial proliferative expansion of pre-formed, antigen-specific Treg cells. This expansion led to an overall increase in the number and relative percentages of Foxp3^IRES-GFP+^ cells as a fraction of total CD4^+^ T cells, as compared to untreated control mice (Fig. 4b and d). Notably, administration of αDCIR2-HA_109–117_ mAb lead to at least 2.5-fold increases in numbers of natural Foxp3^IRES-GFP+^ Treg cells selectively in the spleen, as compared to untreated and αDEC-205-HA_109–117_ mAb-treated mice. This increase was much less notable in other peripheral lymphoid tissues, such as mesenteric or subcutaneous lymph nodes. This observation could account for dominant tolerance in DCIR2^+^ DC-targeted EAE protection of SJL/J mice, as observed in the cell transfer studies described above.

## Discussion

Studies describe multiple methods of administering antigens to induce tolerogenic mechanisms (Miller et al., 2007), but the problem with repeated administration of antigens is its ability to induce fatal autoimmune responses in mice (Pugliese et al., 2001). This problem can be circumvented by direct targeting of minute amounts of antigens to immature DC in vivo*,* allowing the antigen to be delivered efficiently and raising the probability of a tolerogenic response, while lowering the probability of adverse reactions.

It has previously been known that DCIR2+ DC induce tolerance by expansion of existing Tregs (Yamazaki et al., 2008; Kretschmer et al., 2006; Yamazaki & Steinman, 2009), but it was unclear whether targeting the receptor with a fusion antibody in the EAE mouse model would cause immune tolerance, and if so, what the mechanism of this tolerance would be.

One notable difference between the MOG_35–55_ model used in previous studies and PLP_139–151_ induced EAE used in the present study, is that preimmunization of animals with large doses of MOG_35–55_ in the absence of adjuvants is protective against EAE, whereas similar preimmunization with PLP_139–151_ is not (Kuchroo et al., 2002). The striking amelioration of EAE by preimmunization with the αDCIR2-PLP_139–151_ fusion mAb suggests that the binding of fusion mAb to the DC receptors alters the response of these cells to antigen. The lack of protection caused by free PLP_139–151_ preimmunization in SJL/J mice indicates that protection conferred by the fusion mAb is likely due to DC targeting. In addition, while the SJL/ PLP_139–151_ model is a relapsing-remitting model of MS, we could not compare the rate of relapse between different treatment groups due to high mortality in the control group.

A dominant suppressive mechanism of immunological tolerance probably plays a role in EAE amelioration in mice preimmunized with αDCIR2-PLP_139–151_ fusion mAb. We did observe that splenocytes adoptively transferred from αDCIR2-PLP_139–151_ mAb treated mice efficiently prevented EAE induction in recipients, suggesting that the regulatory phenotype was mediated by a type of immune cell (Fig. 1). However, as we couldn’t track antigen specific T cells within the polyclonal T cell repertoire, we could not assess conversion to Tregs.

Our subsequent experiments appears to indicate that the amelioration of EAE by the αDCIR2-PLP_139–151_ fusion mAb results at least partly from a block of early antigen-specific T cell production in the peripheral lymphoid organs. The reduced proportions of IFN-γ- and IL-17-producing pathogenic T cells in preimmunized mice supports this hypothesis (Fig. 2). It is likely that both deletion and induction of an anergic phenotype in pathogenic T cells contributes to αDCIR2 mAb mediated amelioration of EAE.

To assess how this phenotype may come about, we tracked antigen-specific Thy1.1^+^ T cells transferred into αDCIR2 or αDEC-205-HA_109–117_ fusion mAb treated mice. Treatment with αDCIR2-HA_109–117_ mAb initially resulted in somewhat increased proliferation of Thy1.1+ T cells (Fig. 4a and c), but by day 14, essentially all the cells were deleted in these mice. In contrast, on day 14, significant populations of Thy1.1+ T cells were still detectable in mice that had received the same amount of αDEC-205-HA_109–117_ fusion antibody. This important finding may indicate that a primary mechanism of EAE amelioration by αDCIR2 treatment is T cell deletion. To confirm this finding, we quantified Foxp3 cells in αDCIR2 treated mice and found an insignificant increase in Foxp3 induction on day 7 after transfer (Fig. 3d), followed by efficient deletion of all cells on day 14 (Fig. 3e). It should be noted that this observation is in contrast to αDEC-205 studies, which found a prominent role for regulatory T cells in EAE amelioration.

We also tested whether the low induction of Foxp3^+^ cells in the Thy1.1^+^ T cell transfer experiment was due to a failure of Treg conversion or expansion by transferring a small number of TCR transgenic CD4 + CD25 + Foxp3+ Treg cells able to recognize an HA peptide into immunocompetent congenic recipients. The recipient mice were subsequently injected with either αDEC-205-HA_109–117_ or αDCIR2-HA_109–117_ fusion antibodies. Interestingly, both αDEC-205-HA_109–117_ (Fig. 3c) and αDCIR2-HA_109–117_ fusion mAb (Fig. 3d) promote proliferative expansion of antigen-specific Treg cells. Thus, both fusion mAbs are able to induce Treg expansion, suggesting that the low levels of Treg induction in the Thy1.1+ positive T cell transfer experiment are due to a failure to induce Treg conversion.

These observations confirm the recently proposed difference between DEC205 expressing DCs and DCIR2 expressing DCs, where only DEC-205^+^ cells induce Tregs. (Yamazaki et al., 2008; Kretschmer et al., 2005; Yamazaki & Steinman, 2009; Liu et al., 2013) Our results also suggest that targeting of DEC-205+ DCs is able to induce Treg expansion as well as Treg conversion. Presumably, since both fusion mAbs are able to efficiently ameliorate disease, either Treg induction has a minor role in EAE amelioration or another effect of the αDCIR2 antibody is able to compensate for its lack of ability to efficiently induce Tregs.

Another interesting observation to emerge from our study is that amelioration of EAE triggered by αDCIR2 pre-treatment (Fig. 1a) requires immature DC, since when MPLA was administered together with the αDCIR2-PLP_139–151_ (Fig. 1b) there was no protective effect. These mice developed severe paralysis compared to controls that were only administered PLP_139–151_. This clearly suggests that targeting immature DC is necessary for programming tolerogenic DC and is consistent with current thinking about DC biology (Lutz, 2012). Immature DCs do not express co-stimulatory markers and antigen presentation by these cells will tolerize T cells and induce anergy.

Our study contains several technical caveats that need to be considered when interpreting the results. Chief among them is differences in efficiency of EAE induction between different animals. This could be a result of the variation in the reagents that were used for disease induction, or even the health and prior environmental exposure of different mouse cohorts used. It is important to note that while there were differences between induction efficiency between experiments, within each individual experiment, the protective effect of the fusion mAb was significant.

Another technical caveat is the slight amelioration of EAE observed as a result of pre-treatment with isotype controls. This effect has been documented previously (Stern et al., 2010). While the mechanism of this action is unclear, it might be due to cross-reactivity between the isotype antibodies and immature DC, or phagocytosis and presentation of antigen by macrophages or other APC. The isotype-only controls are therefore a more stringent control for EAE amelioration than untreated mice. In the present study, the dramatic amelioration of EAE compared to all controls illustrates the potency of αDCIR2-PLP fusion mAbs.

Additionally, it is worthwhile to note the lack of available means to identify PLP_139–151_ specific T cells, necessitating a change of antigen to explore the mechanism of the fusion mAb. It would be beneficial to evaluate the PLP_139–151_ specific T cells in an SJL/J mouse once a method of identifying PLP_139–151_ specific T cells becomes available. However, it is reassuring that many of the results obtained using the HA antigen were consistent with what was previously known about the action of αDEC-205 antibodies. Therefore, it appears that HA-specific T cells are likely to be a good alternative model for exploring the effect of fusion antibodies, if desired transgenic animals are unavailable.

## Conclusion

In summary, our results indicate that using antibodies other than αDEC-205 to target various subsets of conventional DCs can lead to amelioration of EAE. αDCIR2-PLP fusion mAbs are able to induce expansion of Tregs while having less influence over converting them than DEC-205, yet amelioration of disease is not affected. Thus, induction of Tregs may not be essential for the protective effects of αDCIR2 fusion antibodies. In contrast, deletion of pathogenic T cells seems to play an important role in the mechanism of action of αDCIR2 fusion antibodies. Further analysis of the response of the immune system to different fusion mAbs can give us insight into the function of DC and the mechanisms of tolerance induction.

## Additional file

Additional file 1: **Figure S1.** Flow cytometric isolation of conventional CD4^+^ T cells and CD4^+^Foxp3^+^ Treg cell populations that possess the same antigen specificity. CD4^+^ T cells with transgenic expression of the TCR-HA_109–117_ were identified in peripheral lymphoid tissues of (**A**) TCR-HA_109–117_ x Foxp3-GFP mice and (**B**) Pgk-HA x TCR-HA_109–117_ x Foxp3-GFP mice using the clonotypic antibody 6.5. Pre-sort (top) and post-sort (bottom) analysis of (**A**) conventional CD4^+^TCR-HA^+^ T cells with a naïve CD62L^high^CD25^−^Foxp3^−^ phenotype, and (**B**) CD4^+^Foxp3^+^CD25^+^TCR-HA^+^ Treg cells. Numbers in dot plots indicate the percentages of cells within the respective gate or quadrant. (DOCX 107 kb)

## Abbreviations

APC: Antigen presenting cells
DC: Dendritic cells
EAE: Experimental autoimmune encephalomyelitis
MS: Multiple sclerosis
PLP: Proteolipid protein
