# Supplementary figures and images for: Targeting DEC-205−DCIR2+ dendritic cells promotes immunological tolerance in proteolipid protein-induced experimental autoimmune encephalomyelitis
Source: Mol Med. 2018 May 3;24:17. doi: 10.1186/s10020-018-0017-6 (PMC6016871; doi:10.1186/s10020-018-0017-6)

**Additional file 1: Figure S1**


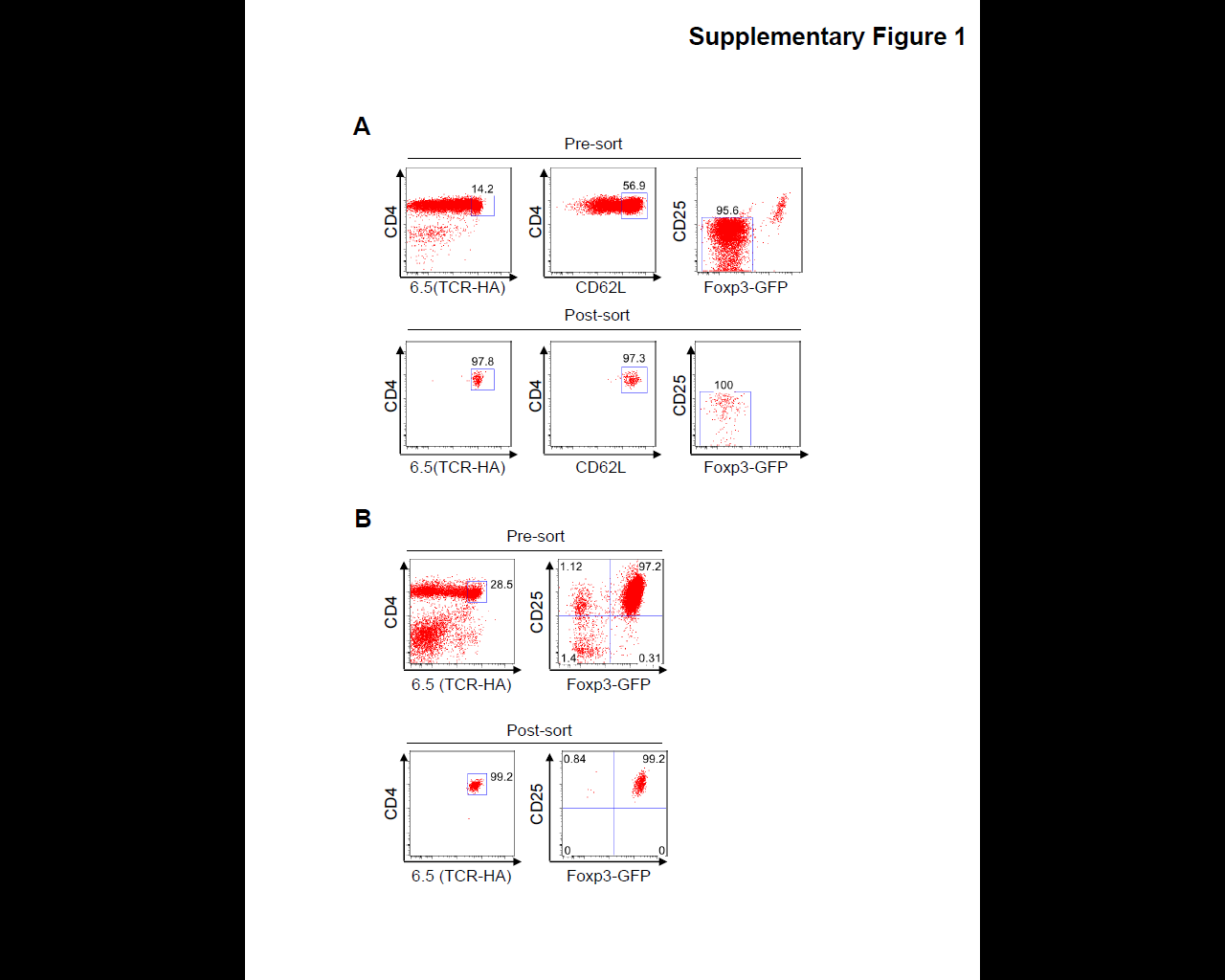

Supplement: Supplementary file 1 — Figure S1. Flow cytometric isolation of conventional CD4+ T cells and CD4+Foxp3+ Treg cell populations that possess the same antigen specificity. CD4+ T cells with transgenic expression of the TCR-HA109–117 were identified in peripheral lymphoid tissues of (A) TCR-HA109–117 x Foxp3-GFP mice and (B) Pgk-HA x TCR-HA109–117 x Foxp3-GFP mice using the clonotypic antibody 6.5. Pre-sort (top) and post-sort (bottom) analysis of (A) conventional CD4+TCR-HA+ T cells with a naïve CD62LhighCD25−Foxp3− phenotype, and (B) CD4+Foxp3+CD25+TCR-HA+ Treg cells. Numbers in dot plots indicate the percentages of cells within the respective gate or quadrant. (DOCX 107 kb) [file 10020_2018_17_MOESM1_ESM.docx]
